# In Situ Preparation of Chlorine-Regenerable Antimicrobial Polymer Molecular Sieve Membranes

**DOI:** 10.3390/molecules29132980

**Published:** 2024-06-23

**Authors:** Yu Zhang, Yiduo Qian, Yuheng Wen, Qiudi Gui, Yixin Xu, Xiuhong Lu, Li Zhang, Wenliang Song

**Affiliations:** 1 Shanghai Key Laboratory of Molecular Imaging, School of Pharmacy, Shanghai University of Medicine and Health Sciences, Shanghai 201318, China; qianyiduo@stu.sumhs.edu.cn (Y.Q.); zhangl_21@sumhs.edu.cn (L.Z.); 2 State Key Laboratory of Molecular Engineering of Polymers, Fudan University, Shanghai 200433, China; 3 School of Materials and Chemistry, University of Shanghai for Science and Technology, Shanghai 200093, China

**Keywords:** *N*-halamines, antimicrobial, membranes, 5,5-dimethylhydantoin

## Abstract

Microbial contamination has profoundly impacted human health, and the effective eradication of widespread microbial issues is essential for addressing serious hygiene concerns. Taking polystyrene (PS) membrane as an example, we herein developed report a robust strategy for the in situ preparation of chlorine-regenerable antimicrobial polymer molecular sieve membranes through combining post-crosslinking and nucleophilic substitution reaction. The cross-linking PS membranes underwent a reaction with 5,5-dimethylhydantoin (DMH), leading to the formation of polymeric *N*-halamine precursors (PS-DMH). These hydantoinyl groups within PS-DMH were then efficiently converted into biocidal *N*-halamine structures (PS-DMH-Cl) via a simple chlorination process. ATR-FTIR and XPS spectra were recorded to confirm the chemical composition of the as-prepared PS-DMH-Cl membranes. SEM analyses revealed that the chlorinated PS-DMH-Cl membranes displayed a rough surface with a multitude of humps. The effect of chlorination temperature and time on the oxidative chlorine content in the PS-DMH-Cl membranes was systematically studied. The antimicrobial assays demonstrated that the PS-DMH-Cl membranes could achieve a 6-log inactivation of *E. coli* and *S. aureus* within just 4 min of contact time. Additionally, the resulting PS-DMH-Cl membranes exhibited excellent stability and regenerability of the oxidative chlorine content.

## 1. Introduction

In recent years, the emergence of multiple bacterial infectious diseases caused by microorganisms has posed a significant threat and instilled a sense of fear in humanity [1,2,3]. Microbial contamination in the air and on surfaces significantly contributes to the transmission and outbreak occurrences of bacterial infectious disease [4,5]. Bactericidal gas and spray are commonly used disinfectants for killing most microorganisms [6]. Nevertheless, these water-soluble disinfectants suffer from some drawbacks due to their instability in aqueous solutions and their propensity to react with organic contaminants in water, leading to the generation of deleterious byproducts [7]. Consequently, there is a significant need for disinfectants that are both insoluble and highly effective, addressing the growing concern over emerging infectious diseases. Numerous studies have reported the incorporation of bactericidal agents into a wide range of polymeric films, such as chitosan, cellulose, polylactic acid, and polyethylene terephthalate, etc. [8,9,10,11,12,13]. These polymeric films have been innovatively enhanced with a suite of bactericidal additives, such as metal-based nanoparticles, quaternary ammonium salts, phosphonium salts, *N*-halamines, biguanides, and isothiazoles, to bolster their antimicrobial properties [14,15,16,17,18,19,20,21]. Among them, *N*-halamines have drawn considerable research attention for their outstanding antimicrobial efficacy, high stability in water, exceptional durability and regenerability upon exposure to washing cycles, and their non-corrosive nature, as well as being nontoxic to humans [22,23]. *N*-Halamines refer to organic compounds that contain one or more N-X (X being Cl, Br, I) covalent bonds, formed by the reaction of precursor compounds that contain amino, amide, or imide groups with oxidizing agents, such as hypochlorites [24,25]. The antimicrobial property of *N*-halamines stems from the oxidative characteristics of halogen atoms within the N-X bond, which possess a positive charge. The consensus is that the antimicrobial action of *N*-halamines is primarily due to the behavior of these oxidized halogen atoms. *N*-halamines discharge potent oxidative halogen cations, which compromise the cell membrane, infiltrate microbial cells, and disrupt the function of cellular enzymes and metabolic pathways, ultimately resulting in bacterial demise without inducing microbial resistance [26].

Among the diverse family of *N*-halamines, 5,5-dimethylhydantoin (DMH) and its derivatives emerges as one of the most widely utilized antimicrobial agents [27,28]. Dong and co-authors reported 1,3-dichloro-/1,3-dibromo-5,5-dimethylhydantoin-containing antibacterial polymeric fibers via an one-step electrospinning method [29]. Through the combined mechanisms of contact and release actions, the as-spun fibers effectively kill bacteria and retain their antibacterial properties even after five recycle experiments. Ding also innovated by creating novel superhydrophilic inorganic-based DMH nanofibrous membranes, which were fabricated through a synergistic approach that combined electrospinning with sol-gel processing [30]. The superhydrophilic membranes demonstrated exceptional rechargeability, a robust resistance to water swelling, and superior antimicrobial efficacy, making them well-suited for the dynamic disinfection of water contaminated with bacteria. Chylińska reported biocidal CS films by incorporating DMH and its hydantoin derivatives to the CS matrix [31]. These biocidal chitosan films exhibited promising applications in the fields of biomedicine, pharmaceuticals, and cosmetics. Worley and co-authors prepared antimicrobial cotton fabrics by immersing cotton fabrics in a DMH precursor polymer solution [32,33]. The DMH precursor polymers exhibited insufficient bonding capabilities with cotton fabrics, resulting in their ready detachment in harsh conditions and consequently causing a significant reduction in antimicrobial performance. Consequently, the challenge rests in developing protective materials that are not only homogeneous and covalently bonded, but also stable and innately endowed with aitimicrobial activity. Recently, Zhang pioneered the development of a positively charged and porous material, FePPOP_Hydantoin_, by incorporating 1,3-dibromo-5,5-dimethylhydantoin (BDMH) and iron porphyrin units into a polymer framework, thereby creating a novel disinfectant [34]. They noted that the direct interaction between bacteria and the BDMH units in FePPOP_Hydantoin_ endowed the material with an antibacterial efficiency exceeding 99.999%.

**Figure 1:**
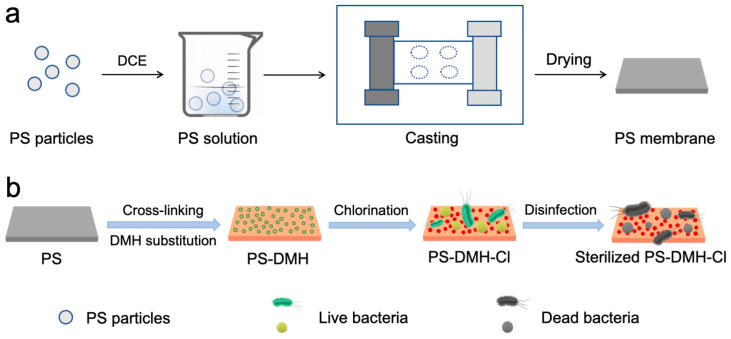
Schematic illustration of the preparation procedure for (**a**) the PS membranes and (**b**) the biocidal PS-DMH-Cl membranes.

In this study, we present a robust strategy for the in situ preparation of chlorine-regenerable antimicrobial polymer molecular sieve membranes through combining post-crosslinking and nucleophilic substitution reaction. As illustrated in Figure 1, the pre-processed polystyrene (PS) membranes were readily cross-linked in situ to prepare hyper-crosslinked porous polymeric membranes (PS-Br) through a Friedel–Crafts reaction. Subsequently, the polymeric *N*-halamine precursors, PS-DMH, were synthesized through a nucleophilic substitution reaction between PS-Br and DMH. After treatment with sodium hypochlorite solution for chlorination, PS-DMH underwent a transformation into its biocidal form, PS-DMH-Cl. A range of experimental parameters were investigated, including the time for cross-linking, as well as the temperature and duration of chlorination. The prepared PS-DMH-Cl membranes were found to feature hierarchical pores and high surface areas, offering ample contact fields for bacteria. An in vitro antimicrobial analysis demonstrated that the PS-DMH-Cl membranes are capable of effectively inactivating both Gram-positive and Gram-negative bacteria. The rational combination of the processable PS membranes together with antibacterial *N*-halamine would result in promising application in water purification systems. More importantly, the strategy outlined boasts the potential for large-scale industrial production and offers the flexibility to be customized in fabricating biocidal products that are specifically designed to meet the diverse needs of various application contexts.

## 2. Results and Discussion

### 2.1. Characterization of Polymeric N-halamine Membranes

**Figure 2:**
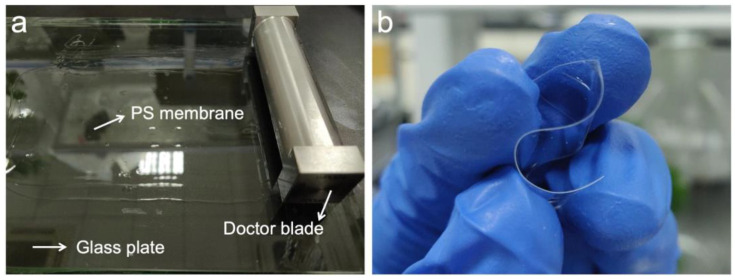
(**a**) Preparation of the PS membrane utilizing a wet film coating applicator. (**b**) The as-prepared PS membrane exhibits excellent flexibility and can be easily bent to accommodate various applications.

PS is a widely used commercial polymer that can be readily processed into membranes by being dissolved in a nonpolar solvent, followed by casting onto an appropriate substrate (Figure 1a and Figure 2a) [35]. The PS membranes are highly flexible, allowing for easy bending (Figure 2b), and its dimensions and shape can be customized to suit specific application requirements. The PS membranes were subjected to a Friedel–Crafts reaction, employing BME as the cross-linker and FeCl_3_ as the catalyst in suit to produce hyper-crosslinked porous polymeric membranes, denoted as PS-Br (Figure 1b). PS-Br was then reacted with DMH via a nucleophilic substitution reaction, yielding the polymeric *N*-halamine precursor, termed as PS-DMH. The initial thickness of the unmodified PS membrane was approximately 822 ± 5.0 μm, and this thickness incrementally increased through sequential modification processes (Table 1). The wettability study was conducted through measurements of the water contact angle (WCA). The WCA of the PS membrane was measured at 91.1 ± 0.3°, which is in close agreement with the values found in the literature, indicative of the membrane’s substantial hydrophilicity (Table 1) [36]. Slight changes were observed following the sequential modifications applied to the surface of the PS membrane. Upon the transformation of the N–H bond in PS-DMH to an N-Cl bond, the WCA of the PS-DMH membrane exhibited a slight increase from 91.3 ± 0.7 to 94.3 ± 0.5. This indicates that the N-halogenation process of the PS-DMH membrane likely has a minimal impact on altering its hydrophilicity [30].

**Figure 3:**
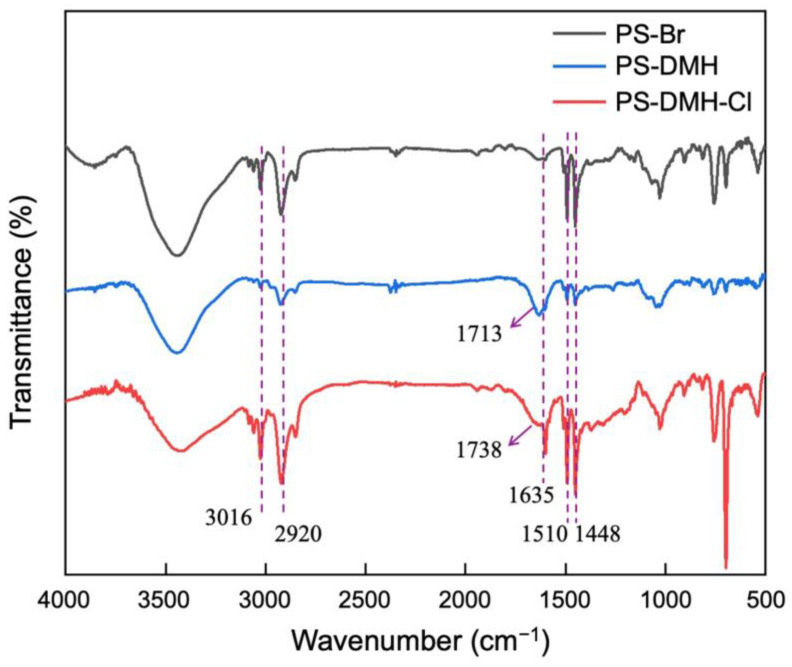
ATR-FTIR spectra of PS-Br, PS-DMH and PS-DMH-Cl membranes.

The ATR-FTIR spectra were recorded to confirm the incorporation of hydantoin moieties (Figure 3). For PS-Br, the characteristic peaks at 3016 and 2920 cm^−1^ were attributed to the C–H stretching vibration of the benzene ring and the carbon skeleton of PS, respectively. The absorption peaks at 1635 and 1510 cm^−1^ corresponded to the stretching vibrations of the C–C and C=C bonds in the benzene ring, respectively, while the peak observed at approximately 1448 cm^−1^ was indicative of the C–H bending vibration in the carbon skeleton of PS. Compared to the spectra of PS-Br, a strong peak observed at 1713 cm^−1^ in the PS-DMH spectra was assigned to carbonyl group of the hydantoin, suggesting the successful incorporation of DMH moieties. After chlorination, this peak shifted to the higher wave number, which is in agreement with the observations reported in earlier studies [37,38]. These collective findings substantiate the successful fabrication of the antimicrobial PS-DMH-Cl membranes.

**Figure 4:**
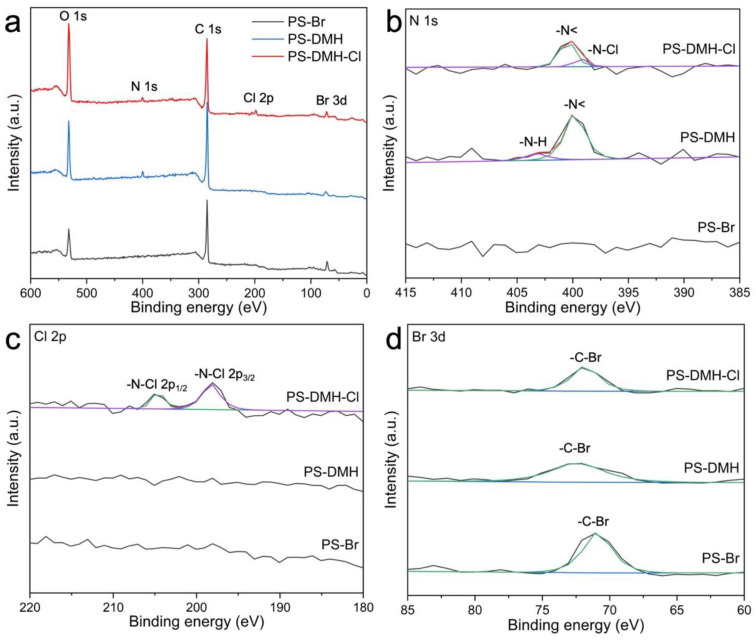
(**a**) XPS survey profiles, and deconvoluted XPS profiles for (**b**) N 1s, (**c**) Cl 2p, and (**d**) Br 3d of PS-Br, PS-DMH and PS-DMH-Cl membranes.

XPS analysis provided further insights into the detailed chemical changes that occurred on membrane surfaces after nucleophilic substitution and chlorination. The PS-Br membrane displayed two main peaks at approximately 284 and 71.1 eV, corresponding to C 1s and Br 3d, respectively (Figure 4a). The PS-DMH membrane exhibited an additional peak around 400 eV, which can be attributed to N 1s (Figure 4b). The N 1s deconvoluted peaks at 403.2 and 399.8 eV were assigned to the covalently bonded amide nitrogen (N–H) and imide nitrogen (–N<) in hydantoinyl groups, respectively, demonstrating the successful nucleophilic substitution reaction with DMH [7]. After chlorination, the PS-DMH-Cl membrane exhibited a new N 1s deconvoluted peak at 399.4 eV, indicative of the formation of N–Cl bonds, while the previously observed peak at 403.2 eV was no longer detectable, suggesting the transformation of hydantoin into *N*-halamine structures. Moreover, the Cl 2p core-level spectra unveiled two additional peaks centered at 203.5 and 199.9 eV, corresponding to Cl 2p_1/2_ and Cl 2p_3/2_, respectively, thereby providing further compelling evidence for these findings (Figure 4c) [37]. Additionally, the Br 3d signals observed in the PS-DMH and PS-DMH-Cl membranes were diminished compared to those of the PS-Br membrane, suggesting that the bromide content was decreased as a result of nucleophilic substitution (Figure 4d). The aforementioned characterization clearly evidenced the successful incorporation of hydantoin moieties and subsequent chlorination.

**Figure 5:**
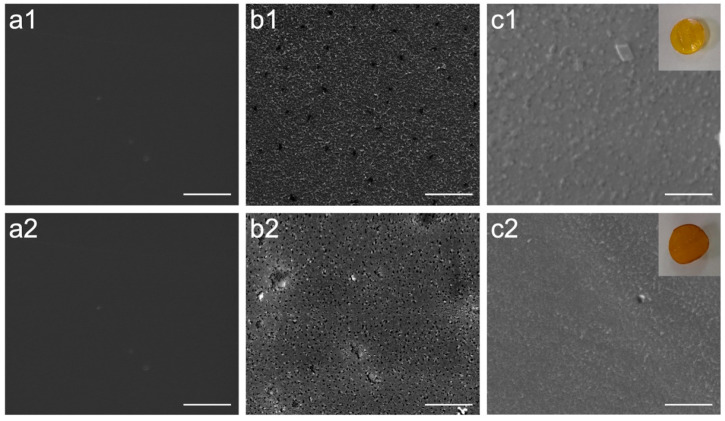
SEM images of (**a1**,**a2**) the pristine PS membranes, (**b1**,**b2**) PS-Br membranes, and (**c1**,**c2**) PS-DMH-Cl membranes. The cross-linking times for samples (**a1**) and (**a2**) are 3 and 9 min, respectively. The images inserted in (**c1**,**c2**) are the as-prepared PS-DMH-Cl membranes. The scar bars are 4 μm.

The surface morphologies of the membranes were characterized by SEM. As shown in Figure 5(a1,a2), the pristine PS membranes revealed a smooth, uniform, and non-porous surface, with their thicknesses being approximately 103 μm and 98 μm, respectively. Upon a cross-linking duration of 3 min, the resultant PS-Br membranes displayed a multitude of noticeable pinholes and diminutive nanoparticles (Figure 5(b1)). Additionally, extending the cross-linking duration to 9 min resulted in the PS-Br membranes displaying a hierarchical surface, featuring a high density of micropores alongside a modest quantity of mesoporous regions (Figure 5(b2)). Increasing the cross-linking reaction time further allows the reaction to proceed more thoroughly. However, an excessively prolonged cross-linking time can lead to the degradation of the cross-linked membrane. Following the nucleophilic substitution and chlorination processes, it is likely that the hierarchical pores were obscured as they became obstructed by the hydantoin groups (Figure 5(c1,c2)). Moreover, the chlorinated PS-DMH-Cl membranes displayed a multitude of humps, which significantly increased the contact area available to counteract bacteria. The thickness of these membranes exhibited negligible variation, with extended cross-linking duration, the integration of hydantoin groups, and chlorination.

**Figure 6:**
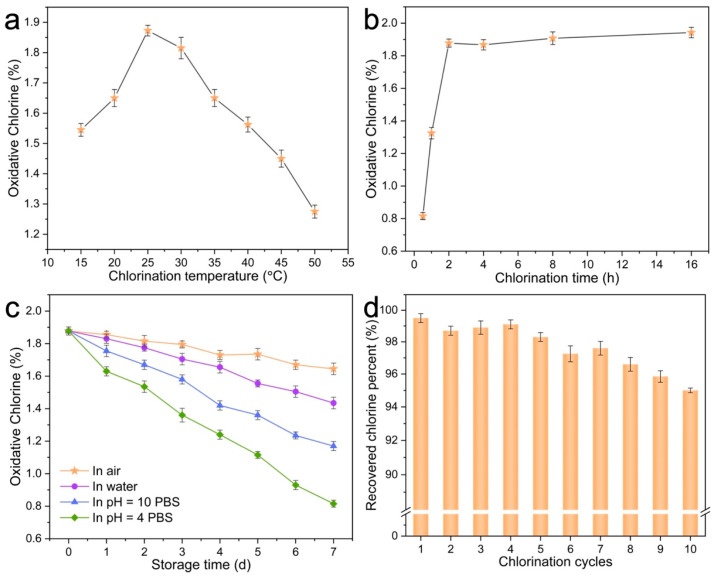
Effect of (**a**) chlorination temperature and (**b**) chlorination time on the oxidative chlorine content in PS-DMH-Cl membranes. (**c**) Stability of the oxidative chlorine content in PS-DMH-Cl membranes under various conditions. (**d**) The regenerability of PS-DMH-Cl membranes.

The antimicrobial efficacy of *N*-halamines is highly dependent on their oxidative chlorine content. Consequently, the effect of chlorination temperature and duration on the oxidative chlorine content of PS-DMH-Cl membranes has been meticulously studied. Figure 6a presents the data obtained from experiments conducted at chlorination temperatures ranging from 15 °C to 50 °C, under a chlorination duration of 2 h. A significant surge in oxidative chlorine content was noted when the temperature was raised from 15 °C to 25 °C, which can likely be ascribed to the accelerated chlorination rate resulting from the increased system temperature. Nevertheless, upon increasing the temperature further from 25 °C to 50 °C, a noticeable reduction in oxidative chlorine content was detected. This trend is likely explained by the enhanced thermal decomposition of both the N-Cl bond and NaClO at the higher temperatures. Figure 6b illustrates the effect of chlorination time on the oxidative chlorine content at 25 °C. As the chlorination time progressed, there was a gradual increase in the oxidative chlorine content initially, which then approached a steady state after 2 h. This phenomenon could be a result of saturation behavior, implying that chlorination is capable of nearing the saturation point within 2 h, thus obviating the need for a prolonged chlorination duration. Therefore, it becomes evident that the PS-DMH-Cl membranes synthesized at 25 °C for a duration of 2 h represent the best option, characterized by their highest oxidative chlorine content.

Given the propensity for *N*-halamines to decompose readily, the enduring stability of PS-DMH-Cl membranes is an essential factor for their practical application. After 7 days of storage in air, a substantial 91.4% of the initial oxidative chlorine content was still retained (Figure 6c). The oxidative chlorine content of the PS-DMH-Cl membranes reduced to 78.9% upon immersion in water. Remarkably, the oxidative chlorine content of the PS-DMH-Cl membranes demonstrated a pronounced pH-dependent behavior, with the content markedly decreasing to 64.5% at a pH of 10 and further to 43.1% at a pH of 4 when maintained in PBS. This phenomenon may be reasonably attributed to the gradual decomposition of *N*-halamines, which continuously release oxidative chlorine ions into the water through a hydrolysis reaction, a process that can be facilitated under both acidic and basic conditions. The facile rechargeability of *N*-halamines is a distinct advantage over other antimicrobial agents, which is crucial for antimicrobial performance. As illustrated in Figure 6d, after 10-cycle chlorination–dichlorination treatment, the oxidative chlorine content in the PS-DMH-Cl membranes exhibited commendable stability, implying their strong regenerative performance was enabled by a simple rechlorination.

### 2.2. Antimicrobial Activity of Polymeric N-halamine Membranes

**Figure 7:**
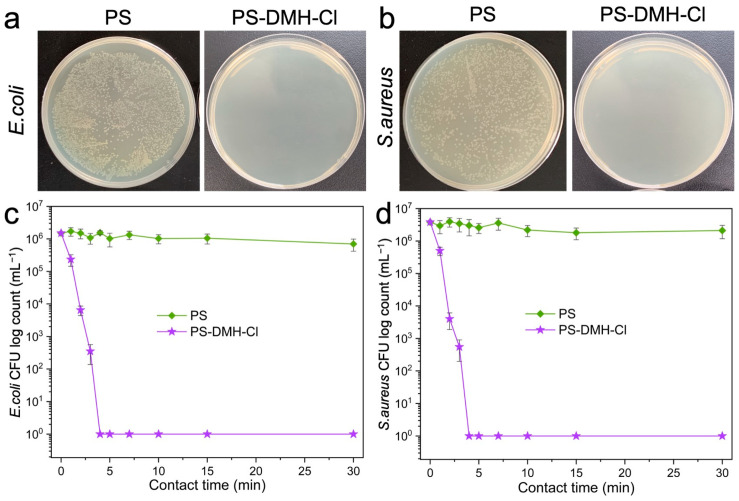
Photographs showing the bacterial culture plates of (**a**) *E. coli* and (**b**) *S. aureus* after treatment with PS and PS-DMH-Cl membranes. Antimicrobial kinetics of PS-DMH-Cl membranes against (**c**) *E. coli* and (**d**) *S. aureus*.

The release of oxidative Cl^+^ from the *N*-halamine structures within PS-DMH-Cl membranes could deactivate bacteria. The Gram-negative bacteria *E. coli* and Gram-positive bacteria *S. aureus* were used as two models, representing pathogenic bacteria in plate counting measurements to assess the antimicrobial efficacy. As presented in Figure 7a,b, significant bacterial proliferation, manifesting as dense clusters of small, white dots, was evident on the plates treated with PS membranes. On the contrary, no bacterial survival was detected on the PS-DMH-Cl membrane-treated plate after 60 min. An antimicrobial kinetics analysis demonstrated that PS-DMH-Cl membranes exhibited remarkable efficacy in eliminating the 6 log CFU of both *E. coli* and *S. aureus* within a mere 4 min of contact (Figure 7c,d). Conversely, the reduction in *E. coli* and *S. aureus* on PS membranes was negligible.

**Figure 8:**
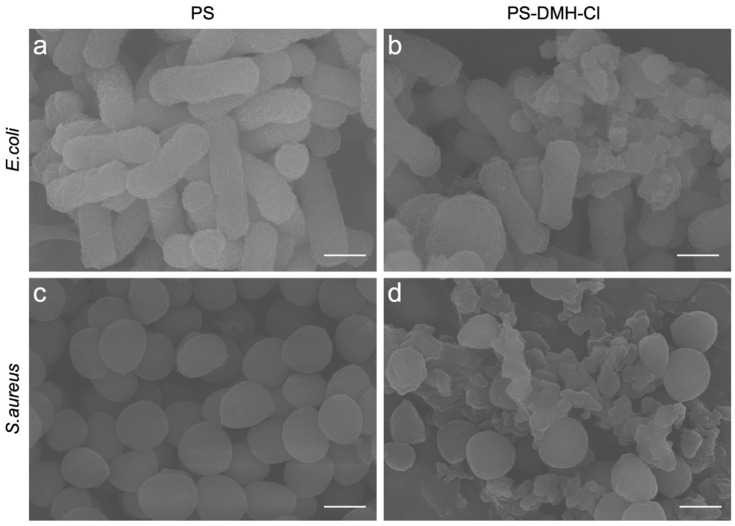
SEM images of (**a**,**b**) *E. coli* and (**c**,**d**) *S. aureus* in contact with PS and PS-DMH-Cl membranes. The scar bars are 1 μm.

To thoroughly understand the antibacterial mechanism of PS-DMH-Cl membranes, the morphological changes of bacterial cells were further investigated. Figure 8 shows the SEM images of *E. coli* and *S. aureus* after treatment with the PS and PS-DMH-Cl membranes. The *E. coli* treated with the PS membrane exhibited a quasi-rod-like shape and a relatively smooth, integrated appearance, consistent with the typical morphology of intact *E. coli* (Figure 8a). In contrast, the *E. coli* treated with the PS-DMH-Cl membrane displayed a noticeably rugged cell wall with several irregular holes (Figure 8b). In the case of *S. aureus*, the bacterial cells treated with the PS membrane showed a grape-like shape with a notably smooth membrane (Figure 8c). In contrast, the *S. aureus* cells treated with the PS-DMH-Cl membrane experienced the leakage of cellular contents and displayed signs of atrophy (Figure 8d). Based on these findings, the oxidative Cl^+^ ions from the PS-DMH-Cl membrane could dissociate and transfer to the bacteria, thereby inactivating them and causing damage to their cellular membranes [30,37,39].

## 3. Experimental Section

### 3.1. Materials

Polystyrene (PS, *Mw* = 192,000), 1,2-dichloroethane (DCE), 5,5-dimethylhydantoin (DMH), and bromomethyl methyl ether (BME) were purchased from Sigma-Aldrich. Sodium hypochlorite (NaClO), potassium hydroxide (KOH), sulfuric acid (H_2_SO_4_), sodium thiosulfate (Na_2_S_3_O_3_), potassium iodide (KI, 99%), anhydrous ferric chloride (FeCl_3_, 98%), ethanol (99%), and phosphate-buffered saline (PBS) were purchased from Shanghai Aladdin Reagent Co., Ltd., Shanghai, China.

### 3.2. Characterization

An Attenuated total reflection-Fourier transformed infrared (ATR-FTIR) spectrophotometer (Thermo Fisher Scientific Co., Ltd., Stoughton, MA, USA) was used to study the chemical structure of the films. The as-obtained membranes were directly scanned in the range of 4000–400 cm^−1^ at a resolution of 4 cm^−1^ [40]. X-ray photoelectron spectroscopy (XPS) measurements were carried out employing a Theta Probe angle-resolved XPS system, which utilized monochromatized Al Kα X-ray radiation. The microscopic morphologies of the as-obtained membranes were evaluated using HITACHI/TM-1000 (Shimadzu, Tokyo, Japan) scanning electron microscope (SEM). The films were affixed to aluminum stubs using double-sided adhesive carbon tape and subsequently coated with a thin gold layer through sputtering.

### 3.3. Preparation of PS Membrane

A total of 17.35 g of PS was added to a conical flask containing 50 mL of DCE. The flask was maintained in an ultrasonic shaker until the PS completely dissolved. An appropriate volume of PS solution was evenly spread onto a 20 × 30 cm glass plate using a wet film coating applicator. After 10 min, the evaporation of the DCE solvent resulted in the formation of a membrane with a defined thickness on the glass plate. This membrane could subsequently be completely peeled off as a transparent and uniform PS membrane using tweezers. The desired regular shape can be obtained by using a mold prior to drying or employing cutting tools post-drying.

### 3.4. Fabrication of Polymeric N-halamine Precursor (PS-DMH)

**Scheme 1:**
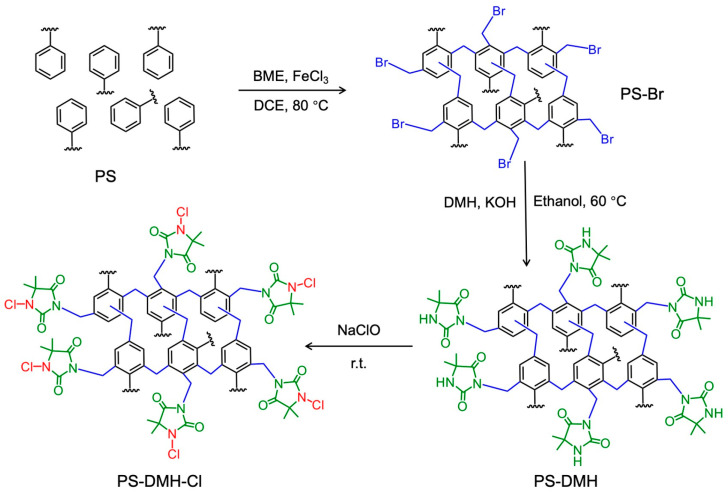
Synthesis pathways of PS-DMH-Cl membranes.

PS-DMH membrane was modified following the protocols described in previously published literatures [35,41,42]. Briefly, 0.5 mL of BME and 1 g of FeCl_3_ were added into a round flask containing 20 mL of DCE; the mixture was then stirred vigorously in an ice bath (Scheme 1). The PS membrane was placed into the flask and subjected to a reaction at 80 °C, under a nitrogen atmosphere, for a predetermined duration (3 min, 9 min). The obtained crosslinked membrane underwent multiple ethanol washes to ensure thorough cleansing, generating hyper-crosslinked porous polymeric membranes; PS-Br. A total of 0.1 g DMH and 0.043 g KOH were introduced to a flask containing 10 mL deionized water and then stirred continuously until the solids were completely dissolved. To the aforementioned solution, 4 mL of ethanol and PS-Br were introduced, and the mixture was subjected to a reaction at 60 °C for 6 h, ultimately yielding a polymeric *N*-halamine precursor, PS-DMH.

### 3.5. Chlorination and Analytical Titration

The PS-DMH membrane was soaked in a 10 wt% NaClO solution, which was stabilized at a pH of 7, and the immersion occurred at room temperature for a period of 1 h. Subsequently, the obtained PS-DMH-Cl membrane was thoroughly washed with deionized water and allowed to air-dry. The amount of active chlorine in the PS-DMH-Cl membrane was detected through the iodometric/thiosulfate titration method [43,44]. A total of 0.1 g PS-DMH-Cl membrane was added to a beaker containing 30 mL of ethanol. A total of 0.2 g of KI and 5 mL 0.04 N H_2_SO_4_ solution were introduced and the mixture was stirred at room temperature for 20 min. Then, 0.01 N of Na_2_S_3_O_3_ solution was used to titrate the mixture. The oxidative chlorine contents (ppm) of PS-DMH-Cl was calculated according to the following equation:

Cl^+^ = N × *V* × 35.45 × 100/(2 × *W*)

where Cl^+^ represents the chlorine loading, N and *V* are the concentration and volume of Na_2_S_3_O_3_ solution, *W* is the weight of PS-DMH-Cl.

### 3.6. Antimicrobial Activity of PS-DMH-Cl

The antimicrobial activity of the PS-DMH-Cl membrane were evaluated using *Escherichia coli (E. coli)* and *Staphylococcus aureus (S. aureus)* as model organisms in a standard plate count assay [45,46]. A total of 1 × 1 cm of PS-DMH-Cl membranes were cultured with bacteria suspensions (1 × 10^6^ CFU mL^–1^) at 37 °C for a variety of durations. A 100 μL aliquot of the co-culture underwent serial dilution in PBS. A total of 10 μL of the diluted co-cultures were evenly spread onto nutrient agar plates and incubated at 37 °C for 12 h. The antimicrobial activity was assessed by counting the number of surviving colonies. All experiments were repeated in triplicate. To evaluate the regenerability of the PS-DMH-Cl membrane, 100 mL of 0.01 N Na_2_S_3_O_3_ solution was added to completely consume the unreacted active chlorine. The membrane was recharged through chlorination as previously detailed.

## 4. Conclusions

In summary, we developed a facile strategy for the fabrication of chlorine-regenerable antimicrobial polymer molecular sieve membranes via in situ post-crosslinking and nucleophilic substitution reaction. DMH groups were covalently integrated into to the cross-linking PS membranes through a simple nucleophilic substitution reaction. The synthesized polymeric *N*-halamine precursors, PS-DMH, converted their hydantoinyl groups into *N*-halamine structures during the chlorination process, leading to the formation of their bactericidal form, PS-DMH-Cl. ATR-FTIR and XPS measurements verified the successful construction of PS-DMH-Cl membranes. The morphology analysis revealed that the chlorinated PS-DMH-Cl membranes displayed a rough surface with a multitude of humps, which significantly increased the contact area available to inactivate bacteria. The optimal conditions for achieving the maximum oxidative chlorine content in the PS-DMH-Cl membranes were carefully selected to be 25 °C and a duration of 2 h. Antimicrobial kinetics analysis suggested that PS-DMH-Cl membranes demonstrated outstanding efficacy in eliminating the 6 log CFU of both *E. coli* and *S. aureus* within a mere 4 min of contact. The PS-DMH-Cl membranes exhibited commendable stability and the ability to be regenerated easily through a simple rechlorination process. Given their unique properties, these chlorine-regenerable antimicrobial PS-DMH-Cl membranes present promising opportunities for use in water purification systems. More importantly, considering the diversity of available polymer species, the in situ cross-linking method can be adapted to develop various types of polymer membranes embedded with specific antimicrobial agents.

## Figures and Tables

**Table 1:** The thickness and WCA of PS, PS-Br, PS-DMH and PS-DMH-Cl membranes.

	PS	PS-Br	PS-DMH	PS-DMH-Cl
Thickness (μm)	822 ± 5.0	827 ± 0.8	835 ± 1.2	836 ± 6.3
WCA (°)	91.1 ± 0.3	93.7 ± 0.6	91.3 ± 0.7	94.3 ± 0.5

## Data Availability

The data presented in this study are available on request from the corresponding author.
